# Habitat associations of bats in a working rangeland landscape

**DOI:** 10.1002/ece3.4782

**Published:** 2018-12-27

**Authors:** Rebecca T. Trubitt, Torre J. Hovick, Erin H. Gillam, Devan A. McGranahan

**Affiliations:** ^1^ School of Natural Resource Sciences—Range Program North Dakota State University Fargo North Dakota; ^2^ Department of Biological Sciences North Dakota State University Fargo North Dakota

**Keywords:** Chiroptera, *Eptesicus fuscus*, land‐use change, *Lasionycteris noctivagans*, *Lasiurusborealis*, *Lasiurus cinereus*, riparian forest, working rangeland

## Abstract

Land‐use change has resulted in rangeland loss and degradation globally. These changes include conversion of native grasslands for row‐crop agriculture as well as degradation of remaining rangeland due to fragmentation and changing disturbance regimes. Understanding how these and other factors influence wildlife use of rangelands is important for conservation and management of wildlife populations. We investigated bat habitat associations in a working rangeland in southeastern North Dakota. We used Petterson d500x acoustic detectors to systematically sample bat activity across the study area on a 1‐km point grid. We identified calls using Sonobat autoclassification software. We detected five species using this working rangeland, which included *Lasionycteris noctivagans *(2,722 detections), *Lasiurus cinereus* (2,055 detections), *Eptesicus fuscus* (749 detections), *Lasiurus* *borealis* (62 detections), and *Myotis lucifugus* (1 detection). We developed generalized linear mixed‐effects models for the four most frequently detected species based on their ecology. The activity of three bat species increased with higher tree cover. While the scale of selection varied between the four species, all three investigated scales were explanatory for at least one bat species. The broad importance of trees to bats in rangelands may put their conservation needs at odds with those of obligate grassland species. Focusing rangeland bat conservation on areas that were treed prior to European settlement, such as riparian forests, can provide important areas for bat conservation while minimizing negative impacts on grassland species.

## 1 INTRODUCTION

Rangelands, or lands on which the dominant natural vegetation is comprised of grasses, forbs, or shrubs, cover approximately 27% of the world's terrestrial surface, but anthropogenic changes have affected the distributions of land cover throughout these systems (Foley et al., 2005; MA, 2005; Society for Range Management, 1998). Human‐driven changes, including altered disturbance regimes, construction of buildings and water sources, and tree planting have altered the distributions of cover on remaining rangelands (Briggs et al., 2005; Fuhlendorf, Engle, Elmore, Limb, & Bidwell, 2012; Lawler et al., 2014; Polasky, Nelson, Lonsdorf, Fackler, & Starfield, 2005). Global patterns of rangeland land‐use and cover change are reflected in the Great Plains of North America, where 49.5% of land has been converted to agricultural or urban uses (Swaty et al., 2011). In addition to continued conversion to row crops, mismanagement and increasing development of energy infrastructure have led to an overall decline in the quality and quantity of grasslands that persist in the region (Allred et al., 2015; Fuhlendorf et al., 2012; Kreuter et al., 2016). Fragmentation and changing disturbance patterns have also prompted changes in the distribution of land cover types (Briggs et al., 2005; Engle, Coppedge, & Fuhlendorf, 2008). In these rangeland landscapes, informed and effective conservation and management requires understanding the variables that impact wildlife distributions and habitat associations (Nielsen, Stenhouse, & Boyce, 2006).

Afforestation is a primary example of changing land cover due to alteration of historic disturbance regimes. Afforestation occurs globally and is particularly rampant in the Great Plains (Engle et al., 2008; Price & Morgan, 2008). Prior to European settlement, tree distribution within North American prairies was limited to areas that were moist and fire inhibited, such as riparian areas and steep draws (Briggs et al., 2005; Engle et al., 2008). However, human development has changed the distribution of trees in rangelands both directly and indirectly. Following droughts and subsequent wind erosion in the 1930s, shelterbelt plantings became widespread, particularly around homesteads and in agricultural areas (Hess & Bay, 2000). Anthropogenic changes such as landscape fragmentation and changes to the fire regimes can also lead to afforestation (Briggs et al., 2005). In an undisturbed landscape, woody cover can increase rapidly, sometimes leading to major regime shifts (Twidwell, Fuhlendorf, Taylor, & Rogers, 2013). Increased woody cover in rangelands promotes generalist and woodland‐adapted species while threatening grassland obligate species (Brennan & Kuvlesky, 2005; Coppedge, Engle, Masters, & Gregory, 2001; Ratajczak, Nippert, & Collins, 2012).

Changing hydrology due to land cover changes can have broad ecological impacts (Gordon, Peterson, & Bennett, 2008; Poff, Bledsoe, & Cuhaciyan, 2006). Agricultural expansion and intensification, dam building, afforestation, and urbanization all cause changes in hydrology, including changes in stream or river flooding and flow patterns, soil water content, and runoff patterns (Gordon et al., 2008; Nilsson & Berggren, 2000; Poff et al., 2006). One important example of ecological change induced by changes to hydrology is the development and destruction of *Populus *riparian forests (Johnson, 1998; Rood & Mahoney, 1990). Riparian forests are important for some wildlife species, including bats, birds, and small mammals (Doyle, 1990; Holloway & Barclay, 2000; Tubbs, 1980). Changing land uses can also alter water distributions at finer scales. For example, agricultural development has led to the draining of many wetlands (Zedler, 2003), and the simultaneous development of dugouts or well‐fed water troughs for cattle water access in working rangeland landscapes, which are managed for both conservation and production goals (Polasky et al., 2005). Although some wildlife use these water sources (Rosenstock, Rabe, O'Brien, & Waddell, 2004; Tuttle, Chambers, & Theimer, 2006), the utility of creating water developments for wildlife conservation is debated (Broyles, 1995).

Bats in rangelands depend on cover types that are actively undergoing change, such as trees and water, and therefore present an interesting case for investigating habitat associations in rangelands. Trees are vital to the life histories of many North American bat species, as they are used during both roosting (Barclay & Kurta, 2007; Carter & Menzel, 2007) and foraging (Prevedello, Almeida‐Gomes, & Lindenmayer, 2017). Similarly, water is important to bats for drinking, particularly because bats experience high evaporative water loss during day roosting (Adams & Hayes, 2008). Additionally, some bat species found in rangelands forage heavily on insects that are found over water sources (Fenton & Bell, 1979). Previous work in rangelands has noted higher bat activity in treed riparian areas (Holloway & Barclay, 2000). The importance of trees to bats in rangelands may put their habitat requirements at odds with many grassland obligate species, which generally respond negatively to woody cover (Brennan & Kuvlesky, 2005; Coppedge et al., 2001; Ratajczak et al., 2012). This potential paradox highlights the importance of understanding bat habitat use in rangelands as bat conservation concerns increase and bat species become listed (U.S. Fish & Wildlife Service, 2015a).

Studies of bat habitat associations in rangelands are necessary because these bat populations provide ecosystem services, face growing threats, and are highly under‐studied (Barclay, 1993; Kunz, Braun de Torrez, Bauer, Lobova, & Fleming, 2011). One of the important ecosystem services that bats provide is insect control (Kunz et al., 2011). Insectivorous bats consume several species of crop pests, an ecosystem service with high value in regions with extensive row‐crop agriculture (Boyles, Cryan, McCracken, & Kunz, 2011; Kunz et al., 2011). North American bats also face growing threats, including white‐nose syndrome, wind energy development, and habitat loss (Arnett & Baerwald, 2013; Frick et al., 2015; Mickleburgh, Hutson, & Racey, 2002). Combating these challenges requires ecosystem‐specific information on bat habitat requirements. Although the bat species inhabiting the Great Plains have distributions covering multiple ecosystems (International Union for the Conservation of Nature, 2015), most of the ecological studies of these species have been conducted in forested areas of their ranges (Amelon, Thompson, & Millspaugh, 2014; Ethier & Fahrig, 2011; Jung, Thompson, Titman, & Applejohn, 1999; Menzel et al., 2005) while relatively little work has been performed on rangeland populations. The relative importance of different landscape features, such as tree patches or water sources, to the habitat selection process may vary between populations inhabiting different ecosystems, as the underlying distributions of these features change (Bolnick et al., 2011). Addressing conservation concerns in rangeland bat populations will require rangeland‐specific information.

In this study, we evaluated the habitat associations of bats in a rangeland landscape in eastern North Dakota. We investigated variables that may provide roosting resources (trees, human‐built structures) and foraging or drinking resources (trees, open water, herbaceous wetlands), and variables that may disrupt access to these resources (roads, row crops) (Adams & Hayes, 2008; Barclay & Kurta, 2007; Carter & Menzel, 2007; Prevedello et al., 2017; Zurcher, Sparks & Bennett, 2010). We evaluated the relationships between bat activity and these variables at both proximate and landscape levels. We hypothesized that both trees and water would be important predictors of bat activity, as has been seen previously in other bat populations (Adams & Hayes, 2008; Amelon, 2007; Ethier & Fahrig, 2011; Holloway & Barclay, 2000). We also expect that variables at both proximate and landscape scales will be important to predicting bat activity in rangelands (Amelon, 2007; Ethier & Fahrig, 2011). This study will help inform the management and conservation of bats in rangelands, and will also aid in balancing the conservation needs of bats with those of grassland obligate species to preserve biodiversity and ecosystem services.

## 2 MATERIALS AND METHODS

### 2.1 Study area

**Figure 1:**
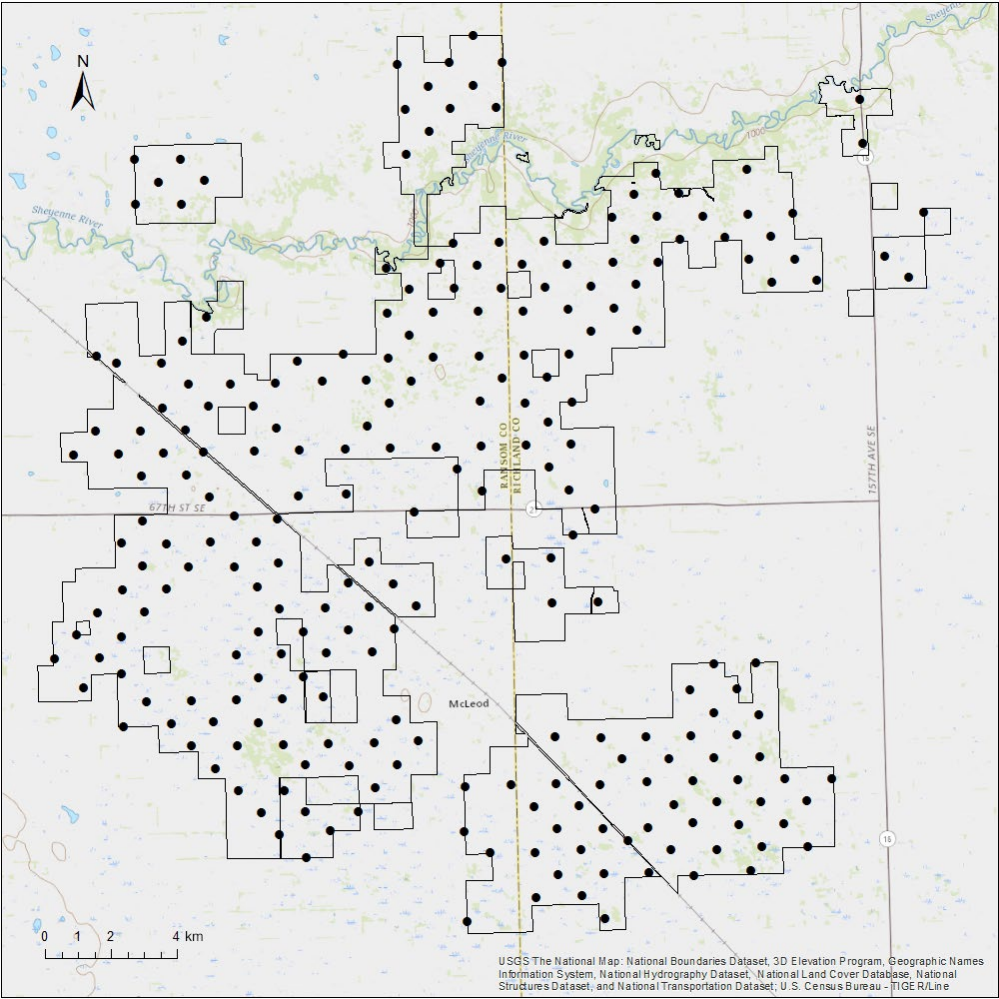
Map of the study area showing study area boundaries (black line) and the 247 points surveyed from May to August of 2016 (black dots). The Sheyenne River runs through the northern portion of the study area (blue line)

This study took place on the United States Forest Service's Sheyenne National Grassland, The Nature Conservancy's Brown Ranch and Pigeon Point Preserve, and North Dakota State University's Albert Ekre Grassland Preserve, which are all located in southeast North Dakota (Figure 1). The total study area is 28,822.12 ha. The climate of this area is temperate, with cold winters and warm summers. During the study period (May to August), monthly average temperatures range from 14.4°C (May) to 22.2°C (July). Most of the yearly precipitation falls during this period, with an average of 310 mm from May to August (NDAWNCenter, 2015). The area is characterized by sandy soils and dunes deposited in the delta of the glacial Lake Agassiz, which form a rolling landscape with a mosaic of wetland and upland grasslands (Knudson, VanLooy, & Hill, 2015). The Sheyenne River flows through the northern part of the study area, and the area is surrounded by mostly agricultural plains (Knudson et al., 2015). The Sheyenne National Grasslands encompass the only remaining tallgrass prairie in the Red River region (Samson et al., 2003), and mixed prairie, prairie wetlands, oak‐aspen savanna, and mixed deciduous forest are also present in the area (Knudson et al., 2015). All the lands within the study area are grazed and managed as working ranches.

### 2.2 Bat survey

We collected acoustic data from 15 May to 14 August 2016 to evaluate bat activity across the Sheyenne National Grasslands and surrounding area. This period encompasses pregnancy, lactation, and early flight of juveniles. Using a random number generator, we randomly selected 237 survey points from a 1‐km point grid across the study area generated using ArcGIS, which contained 304 total points (Figure 1). This systematic approach allowed thorough coverage of the full study area, regardless of landcover type. We used 10 Pettersson d500x bat detectors that were elevated approximately 1.5 m above ground to record echolocation calls (U.S. Fish & Wildlife Service, 2015b). We recorded for three consecutive nights at each survey point, recording from sunset to sunrise each night (Skalak, Sherwin, & Brigham, 2012). The sampling period was extended up to five nights if any precipitation occurred during the recording period, as bat activity can be depressed during rainstorms (Erickson & West, 2002). We retrieved bat detectors after the final night and downloaded recordings which were then analyzed using Sonobat autoclassification software (Sonobat 3.1, MT_Plains package, Arcata, CA). Only calls classified with 95% confidence or higher were accepted as detections, and these calls were manually vetted to ensure accuracy (Barnhart & Gillam, 2014).

### 2.3 Landscape variables

We collected data on both proximate and landscape‐level variables using ArcGIS 10 (ESRI, Redlands, CA) and the R statistical environment (version 3.3.1; R Core Team, 2017). We delineated tree, open water, and crop cover manually in ArcGIS 10 using orthoimagery collected by the National Agriculture Imagery Program (NAIP, 2014). Because herbaceous wetlands were difficult to identify using aerial imagery, we used the National Wetlands Inventory (U.S. Fish & Wildlife Service, 2016) to delineate these areas. Open cover was determined by subtracting the four measured cover class areas from the total buffer area. Land cover was ground truthed during later fieldwork. We then used R to calculate the cover area (m^2^) of these classes and tree patch perimeter length (edge length, m) within 250‐, 500‐, 1,000‐, and 3,000‐m buffers of each sampling point. The ratio of tree patch edge length to tree area was used in modeling to separate the effects of edge from those of cover. We also used R to measure the road density within these buffers, using State and Federal and City and County road datasets from the North Dakota Department of Transportation (NDDOT, 2016a, 2016b). Distances from each sampling point to the nearest live tree, open water source, and human‐built structure were also measured using ArcGIS 10.

### 2.4 Data analysis

We developed generalized linear mixed‐effects models (GLMMs) to assess the relative contributions of each variable to observed bat activity (Appendix S1). Nine models were developed based on the known biology of our study species using the package glmmTMB in R (Brooks et al., 2017; Table 1). Due to overdispersion in the count data, the negative binomial (“nbinom2”) family was used for all models (Brooks et al., 2017). We used number of minutes with a detection as the response variable. Using this measure avoids inflated counts caused by individual behavior, such as bats circling the detector (Miller, 2001). We included detector ID as a random variable to account for differences in detector sensitivity. We assessed the influence of tree, water, wetland, and crop cover, road density, tree edge length, and the proximity of trees, open water, and human‐built structures on bat activity (Table 2). For all of these variables, we used *z*‐scores to allow the comparison of variables with different scales. The *z*‐score is found by subtracting the mean from each observation then dividing by the standard deviation (Hovick et al., 2015). To avoid collinearity between predictor variables, we calculated variance inflation factors (VIFs) using the function “vif.mer” in R (Frank, 2011), and sequentially eliminated covariates with VIFs over 3, the threshold suggested by Zuur, Ieno, and Elphick (2010). This eliminated the open cover class and the 3,000 m scale. As there was also a high level of correlation in landcover variables between scales, models at each scale were considered individually rather than averaged when models at multiple scales were explanatory.

**Table 1:** Generalized linear mixed‐effects models tested for 2016 bat activity data on and near the Sheyenne National Grasslands

Model name	Model variables
Global	TreeDist + WaterDist + StructDist + TreeCover + WaterCover + WetlandCover + CropCover + EdgeRatio + RoadDensity
Landscape	TreeCover + WaterCover + WetlandCover + CropCover + EdgeRatio + RoadDensity
Proximate	TreeDist + WaterDist + StructDist
Landcover	TreeCover + WaterCover + WetlandCover + CropCover
Roost	TreeDist + StructDist^a^ + TreeCover
Tree	TreeDist + TreeCover + EdgeRatio
Water	WaterDist + WaterCover + WetlandCover
Development	StructDist + CropCover + RoadDensity
Null	1

a Structure distance was only included in Roost models for *Lasionycteris noctivagans* and *Eptesicus fuscus*, which have been reported to roost in buildings.

**Table 2:** Summary of measured variables used for modeling 2016 bat activity in and near the Sheyenne National Grasslands

Variable name	Mean	*SD*	Range	Description
TreeDist	186	227	0–1,071	Distance to nearest live tree (m)
WaterDist	398	227	2–1,227	Distance to nearest open water (m)
StructDist	1,795	1,027	10–5,817	Distance to nearest human‐built structure (m)
TreeCover250	7.7%	13.3%	0%–75.8%	Percent tree cover within 250 m of sampling point
TreeCover500	8.1%	11.9%	0%–64.6%	Percent tree cover within 500 m of sampling point
TreeCover1000	8.3%	10.7%	0%–57.9%	Percent tree cover within 1 km of sampling point
WaterCover250	0.2%	0.6%	0%–7.1%	Percent open water cover within 250 m of sampling point
WaterCover500	0.1%	0.5%	0%–6.1%	Percent open water cover within 500 m of sampling point
WaterCover1000	0.2%	0.4%	0%–3.2%	Percent open water cover within 1 km of sampling point
WetlandCover250	10.6%	14.1%	0%–70.7%	Percent herbaceous wetland cover within 250 m of sampling point
WetlandCover500	10.9%	12.0%	0%–55.2%	Percent herbaceous wetland cover within 500 m of sampling point
WetlandCover1000	10.1%	10.4%	0%–43.7%	Percent herbaceous wetland cover within 1 km of sampling point
CropCover250	1.6%	7.4%	0%–44.6%	Percent crop cover within 250 m of sampling point
CropCover500	2.3%	7.9%	0%–49.2%	Percent crop cover within 500 m of sampling point
CropCover1000	4.3%	9.5%	0%–50.2%	Percent crop cover within 1 km of sampling point
EdgeRatio250	0.22	0.28	0–2.0	Tree edge/tree cover ratio within 250 m of sampling point
EdgeRatio500	0.19	0.17	0–1.21	Tree edge/tree cover ratio within 500 m of sampling point
EdgeRatio1000	0.15	0.09	0–0.68	Tree edge/tree cover ratio within 1 km of sampling point
RoadDensity250	103	204	0–974	Meters of road within 250 m of sampling point
RoadDensity500	290	494	0–1,987	Meters of road within 500 m of sampling point
RoadDensity1000	1,143	1,185	0–4,405	Meters of road within 1 km of sampling point

Four species had sufficient detections to use in modeling. We used Akaike's Information Criterion (AIC) and model weights (*w_i_*) to evaluates models at each of three landscape scales (250, 500, and 1,000 m) for each species (Burnham & Anderson, 2002). The explanatory models for each scale were then ranked using AIC to determine the scale of selection for each species (Burnham & Anderson, 2002). The significance of variables included in explanatory models for each species was determined using 95% confidence intervals as calculated by function “confint” in R. We checked for spatial autocorrelation in the residuals of competitive models using Moran's I (Gittleman & Kot, 1990).

## 3 RESULTS

We collected 5,589 detections from five species of North American bats. We detected *Lasionycteris noctivagans* 2,722 times (78% of survey sites), *Lasiurus cinereus *2,055 times (60% of survey sites), *Eptesicus fuscus* 749 times (51% of survey sites), *Lasiurus borealis *62 times (11% of survey sites), and *Myotis lucifugus* 1 time (0.4% of survey sites).

**Figure 2:**
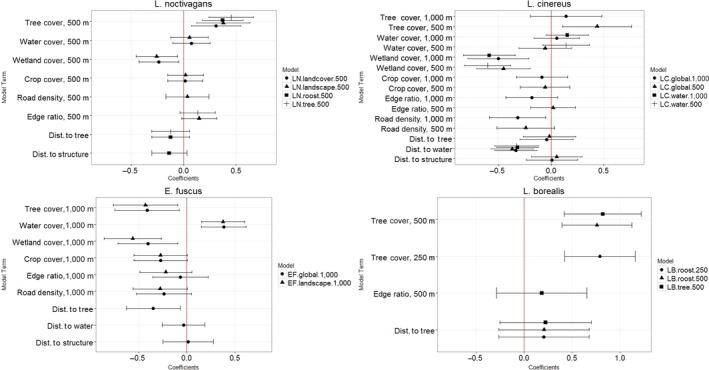
Variable coefficients for the competitive models for the activity levels of *Lasionycteris noctivagans, Lasiurus cinereus, Eptesicus fuscus, *and *Lasiurus borealis *on and near the Sheyenne National Grasslands during the summer of 2016. Coefficient values are indicated by the points, and error bars represent 95% confidence intervals. Positive coefficients indicate that bat activity is positively associated with that variable, and negative coefficients indicate a negative association. Effect size is represented by the magnitude of the variable. The results of all competitive models are presented for each species. For further description of these models, see Table 1

All species responded to tree distributions at either proximate or landscape scales or both. Three species, *L. noctivagans, L. cinereus, *and *L. borealis*, responded positively to tree cover within 500 m (Figure 2). *Eptesicus fuscus* responded negatively to tree cover within 1,000 m, but positively to trees at a proximate level (Figure 2). Bat responses to other variables showed more interspecific variation. Landscape‐level water cover was positively associated with activity of *E. fuscus*, and *L. cinereus* was negatively associated with distance to the nearest open water source (Figure 2). The activity of all species but *L. borealis *was negatively associated with wetland cover (Figure 2). Responses to human infrastructure (crop cover, road density, and distance from human‐built structures) were largely not significant or not included in the most explanatory models. The exception to this is *L. cinereus, *which was negatively associated with road density at the 1,000‐m scale.

Although all four species were associated with landscape‐level variables, the scale at which this association is most explanatory varied. The 250‐m scale was explanatory for *L. borealis, *the 500‐m scale was explanatory for *L. noctivagans, L. cinereus, *and *L. borealis, *and the 1,000‐m scale was explanatory for *L. cinereus *and *E. fuscus *activity.

## 4 DISCUSSION

Bats are important ecosystem service providers, but relatively little is known about their habitat requirements in rangeland landscapes (Barclay, 1993; Chung‐MacCoubrey, 1996; Holloway & Barclay, 2000; Kunz et al., 2011). We analyzed the use of landscape features by bats across three scales in the Great Plains of North America and found that bat activity was positively associated with trees in all four species investigated. This is consistent with findings of previous studies on bats in forested landscapes (Adams & Hayes, 2008; Brigham, 2007; Holloway & Barclay, 2000). The importance of trees highlights the value of riparian forests to bats in rangeland ecosystems (Holloway & Barclay, 2000). These results will be important in guiding conservation efforts for bats in landscapes where trees are commonly viewed as a negative feature, but may serve an important ecological function in the appropriate context (Prevedello et al., 2017).

Higher availability of trees at both proximate and landscape scales was associated with higher bat activity. The activity of three of four species (*L. noctivagans, L. cinereus, *and *L. borealis*) increased as landscape‐level tree cover increased. The fourth species, *E. fuscus, *responded negatively to landscape‐level tree cover but responded positively to tree proximity. Many North American bats, including the four focal species, use trees for roosting (Harvey, Altenbach, & Best, 2011). *L. cinereus *and *L. borealis* are migratory and roost in foliage year‐round, and *L. noctivagans *and *E. fuscus* often roost in cavities and beneath sloughing bark (Harvey et al., 2011). Trees also provide foraging opportunities (Prevedello et al., 2017) and shelter from weather and predators (Verboom & Spoelstra, 1999). For *L. cinereus *and *L. borealis*, the positive responses to landscape‐level tree cover we found in this study have also been reported in forests (Amelon, 2007; Ethier & Fahrig, 2011; Starbuck, Amelon, & Thompson, 2015). However, our results for *L. noctivagans *and *E. fuscus* tree cover responses differ from previous reports from forested regions (Ethier & Fahrig, 2011; Starbuck et al., 2015). In our study, *L. noctivagans* activity was higher at sites with higher tree cover and *E. fuscus* activity was lower in areas of higher tree cover. In previous studies, *L. noctivagans* has responded negatively to higher forest cover (Ethier & Fahrig, 2011), and favors clearcuts and open spaces (Patriquin & Barclay, 2003). Conversely, neutral and positive responses to landscape‐level tree cover have been documented for *E. fuscus* in forests (Ethier & Fahrig, 2011; Starbuck et al., 2015). It has been suggested that some species have thresholds of necessary tree cover (Amelon, 2007). The differences between our results and those from previous studies in forested systems may be due to the overall lower levels of tree cover available in this rangeland landscape. At the local scale, the use of treed areas is modulated by bat morphology, particularly wing morphology (Norberg & Rayner, 1987). Smaller, more maneuverable bats are able to use areas with higher vegetative clutter (i.e., forest interiors), while larger, faster, less maneuverable bats use open areas and edges (Norberg & Rayner, 1987). All four of our focal species are considered open‐area or edge foraging species (Loeb & O'Keefe, 2011), and at proximate scales, positive responses to areas of nonforest have been reported (Amelon, 2007). However, we speculate that the lower levels of tree cover available on rangeland landscapes promote the selection of tree patches rather than open areas that we observed in all focal species.

Of the four focal species, only *E. fuscus *responded positively to landscape‐level open water cover, and only *L. cinereus *responded to water proximity. Our study species have been reported to respond positively to water cover and proximity in eastern deciduous forests of the United States (Amelon et al., 2014; Brooks & Ford, 2005; Dixon, 2012). Water availability is important to bats, as open water provides both drinking and foraging opportunities (Korine, Adams, Russo, Fisher‐Phelps, & Jacobs, 2016). Roosting bats experience high evaporative water loss and replenish 20%–22% of these losses by drinking (Adams & Hayes, 2008). The availability of drinking water is particularly important to lactating individuals, which have been reported to visit drinking holes 13 times more than nonreproductive females (Adams & Hayes, 2008). Open water also provides emergent aquatic insect prey and can concentrate insects (Hagen & Sabo, 2011). Riverine sources may also provide corridors for commuting and migration (Furmankiewicz & Kucharska, 2009). The reported importance of water to bats makes the relative lack of responses to water in this study unexpected. This outcome is likely due to the high levels of open water available in the landscape due to the soils and dunes deposited in the delta of the glacial Lake Agassiz, which form a rolling landscape with a mosaic of wetland and upland grasslands (Knudson et al., 2015). Furthermore, the active ranching practices in the area have introduced many anthropogenic water sources for cattle production. Bats have been documented to use artificial water sources, including dirt, and metal stock tanks (Geluso & Geluso, 2016; Tuttle et al., 2006; Vindigni, Morris, Miller, & Kalcounis‐Rueppell, 2009). It is interesting to note that the activity of three of four focal species was negatively associated with herbaceous wetland cover. We suspect that this is due to the fact that most of the herbaceous wetland cover in our study area was open, cattail dominated swales with little tree cover nearby. The negative association with these wetlands may be more due to the lack of tree cover rather than the wetlands themselves.

Use of acoustic detectors in our study allowed us to cover a broad area efficiently. This thorough spatial coverage of the study area was necessary due to the single‐year study period. Although this approach was needed for our study, the technique does have some drawbacks. Due to the function of echolocation calls, which are used to locate surrounding objects rather than to advertise identity, some calls are not able to be identified to species (Barclay, 1999). This difficulty is compounded when call quality is low. We have addressed this concern by accepting only calls with high‐certainty identifications made by Sonobat (≥95% discrete probability) and hand vetting these calls to ensure accuracy. Several authors recommend a combination of acoustic and mist‐netting techniques for bat surveys to compensate for the shortcomings of each technique (Barclay, 1999; O'Farrell & Gannon, 1999). Although logistical constraints did not allow for a systematic netting effort comparable to our acoustic sampling, opportunistic netting throughout the summer of 2016 confirmed the presence of all four focal bat species in the area, lending credence to our inventory. The use of acoustic survey techniques also left information on age and sex structure and intraspecific variation in landscape use undiscovered. These questions may be productive avenues for future research.

This study shows a strong positive association between tree availability and bat activity in rangeland landscapes. From a range management perspective, the importance of tree cover to bats in rangelands appears to put bat management goals at odds with the needs of obligate grassland wildlife (Brennan & Kuvlesky, 2005; Coppedge et al., 2001; Ratajczak et al., 2012). However, some tree cover existed on rangelands prior to European settlement in areas where sufficient water is available and fire is infrequent, such as riparian areas and steep draws (Briggs et al., 2005; Knopf, Johnson, Rich, Samson, & Szaro, 1988). Riparian forests are small but important parts of the broader rangeland landscape (Knopf et al., 1988). Their importance to bats has been demonstrated both in rangeland and forested systems, and our systematic, landscape‐level approach has reaffirmed the importance of these native, highly tree covered areas (Grindal, Morissette, & Brigham, 1999; Holloway & Barclay, 2000). Riparian forests are also important to other wildlife, including some species of birds and small mammals (Doyle, 1990; Tubbs, 1980). The optimal management of these areas for bats and other wildlife is an important question for future research. Riparian forest dynamics are affected by both stream‐associated and upland‐associated sources of disturbance, including flooding patterns, fire, and grazing (Abrams, 1985; Kozlowski, 2002; Ohmart, 1996; Rood & Mahoney, 1990; Scott, Skagen, & Merigliano, 2003). Understanding the roles of these disturbances, particularly fire and grazing, which are more accessible methods for managers, is important for retaining native structure and disturbance regimes in these important areas.

Our landscape‐level modeling of bat foraging activity in rangelands illustrates the complexity of the factors associated with habitat use in these animals. Relationships between bat activity and landscape features varied between bat species, and several variables, particularly the distributions of trees, were significant predictors of bat activity at both proximate and landscape scales. These results corresponded to findings from rangeland and forested ecosystems (Adams & Hayes, 2008; Amelon, 2007; Ethier & Fahrig, 2011; Holloway & Barclay, 2000). Our approach to modeling was focused on specific variables that we selected a priori, but our models did not explain all of the variation in bat habitat use, illustrating the complexity of modeling habitat use in rangeland landscapes and leaving many questions for future research. For example, future research could focus on the importance of tree patches away from riparian areas, and the importance of tree patch size and tree species composition. Despite the complexity demonstrated, this study shows the importance of trees at both proximate and landscape levels. This in turn highlights the importance of natively treed areas, particularly riparian forests, to rangeland bat populations (Holloway & Barclay, 2000). Focusing management efforts on riparian areas and other fire‐inhibited portions of the landscape can provide important core areas for bat populations that fit into the historical context of the rangeland landscape and complement conservation strategies for grassland obligate wildlife.

## CONFLICT OF INTEREST

None declared.

## AUTHORS’ CONTRIBUTIONS

RTT, TJH, and EHG conceived the ideas and designed methodology; RTT collected and analyzed the data; RTT and TJH led the writing of the manuscript. All authors contributed critically to the drafts and gave final approval for publication.

## DATA ACCESSIBILITY

Data and R code will be freely accessible through Dryad data repository upon publication (https://doi.org/10.5061/dryad.r17q65s).
