# Supplementary material for: Habitat associations of bats in a working rangeland landscape
Source: Ecol Evol. 2018 Dec 27;9(1):598–608. doi: 10.1002/ece3.4782 (PMC6342184; doi:10.1002/ece3.4782)
Supplement: Supplementary file 1 [file ECE3-9-598-s001.docx]

#Chapter 1: Habitat Associations of Rangeland Bats

#Fieldwork took place from mid May to mid August, 2016.

#Response variable is minutes with detection (see Miller, 2001).

#Data file. This file contains Z-scores rather than raw data. Z scores are used so that coefficients from variables with different magnitudes

#can be compared. Field descriptions: "Point"- Survey point number. "Detector" - Bat detector ID. "TimeRec"- minutes the detector was recording.

# "EpfuMin etc"- number of minutes with a detection of that species. Epfu= E. fuscus, Labo=L. borealis, Lano= L. noctivagans, Laci=L.cinereus.

#"TotalMin"- number of minutes with any bat detection. "Long" and "Lat"- longitude and latitude. All of the following variables are Z scores of

#measured variables. The code to measure these variables is included below. Variables that started with P are proximate variables- "distance to" variables.

#Variables starting with L are landscape variables. The Z in the second character indicates that these are Z scores. The third area is the landscape

#feature being measured- trees, water, structures etc. For landscape level variables, the radius that the variable is measured at is listed last in the

#name- these can be 250, 500, 1000 or 3000m radii. Note- "Ratio" is the ratio of tree edge length to tree area, and "Edge" is just the length of the

#tree edge. I used "Ratio" for modelling, as tree edge length is highly correlated with tree area.

act<-read.csv("E:/ResearchR/data/2016_Chapter1ModelVariables.csv")

act<-act[1:237,]

#Packages:

install.packages("lme4")

install.packages("glmmTMB")

install.packages("ggplot2")

install.packages("gridExtra")

install.packages("MASS")

install.packages("ape")

library(lme4)

library(glmmTMB)

library(ggplot2)

library(gridExtra)

library(MASS)

library(ape)

install.packages("pacman")

pacman::p_load(maps, ggplot2, ggmap, broom, rgdal, rgeos,plyr, maptools )

install.packages("raster")

library(raster)

install.packages("sp")

library(sp)

#Step 1: Data exploration

##Density histograms to check distrubution of bat activity data- determines which family/link function is used for generalized linear models.

##The shown code is for total number of calls, but you can also break it down by species.

Ch1Distribution<- ggplot(act, aes(x=TotalMin)) + theme_bw() +

geom_histogram(aes(y=..density..),

binwidth=1,

colour="black", fill="lightgreen") +

geom_density(alpha=.2, fill="#FF6666")

##Use boxplot/Kruskal-Wallis test to test for differences between bat detectors. Determines whether GLM or GLMM is used.

Ch1Det<-ggplot(act, aes(x=Detector, y=TotalMin)) + geom_boxplot()

kruskal.test(act$TotalMin, act$Detector)

#Step 2: GIS code to gather landscape variable data

##Make layers

proj<-readOGR(dsn= "C:/Users/rebecca.trubitt/Desktop/Thesis GIS", layer= "Landcover_Projected")

proj@data$id <- rownames(proj@data)

proj.points <- fortify(proj, region="id")

proj.df <- join(proj.points, proj@data, by="id")

pt<-read.csv("C:/Users/rebecca.trubitt/Desktop/ResearchR/data/Pts_R.csv")

coordinates(pt) = ~x + y

##Make buffers around survey points at 250, 500, 1000, 3000m radii

buff250<-gBuffer(pt186, byid=TRUE, id=NULL, width=250, quadsegs=8, capStyle="ROUND",

joinStyle="ROUND", mitreLimit=1.0)

buff500<-gBuffer(pt186, byid=TRUE, id=NULL, width=500, quadsegs=16, capStyle="ROUND",

joinStyle="ROUND", mitreLimit=1.0)

buff1000<-gBuffer(pt186, byid=TRUE, id=NULL, width=1000, quadsegs=32, capStyle="ROUND",

joinStyle="ROUND", mitreLimit=1.0)

buff3000<-gBuffer(pt186, byid=TRUE, id=NULL, width=3000, quadsegs=96, capStyle="ROUND",

joinStyle="ROUND", mitreLimit=1.0)

proj<- gBuffer(proj, byid=TRUE, width=0) #This command helps fix overlaps in hand-drawn layer

##Clip Landcover shapefile to buffers

int250<-intersect(buff250, proj)

int500<-intersect(buff500, proj)

int1000<-intersect(buff1000, proj)

int3000<-intersect(buff3000, proj)

##Calculate landcover areas and aggregate- gathers numbers for Tree Cover, Water Cover, Crop Cover, and Wetland Cover

area250 <- data.frame(area=sapply(int250@polygons, FUN=function(x) {slot(x, 'area')}))

row.names(area250) <- sapply(int250@polygons, FUN=function(x) {slot(x, 'ID')})

attArea <- spCbind(int250, area250)

agg250<-aggregate(area~attArea@data$Class+attArea@data$Point.No, data=attArea, FUN=sum)

area500 <- data.frame(area=sapply(int500@polygons, FUN=function(x) {slot(x, 'area')}))

row.names(area500) <- sapply(int500@polygons, FUN=function(x) {slot(x, 'ID')})

attArea500 <- spCbind(int500, area500)

agg500<-aggregate(area~attArea500@data$Class+attArea500@data$Point.No, data=attArea500, FUN=sum)

area1000 <- data.frame(area=sapply(int1000@polygons, FUN=function(x) {slot(x, 'area')}))

row.names(area1000) <- sapply(int1000@polygons, FUN=function(x) {slot(x, 'ID')})

attArea1000 <- spCbind(int1000, area1000)

agg1000<-aggregate(area~attArea1000@data$Class+attArea1000@data$Point.No, data=attArea1000, FUN=sum)

area3000 <- data.frame(area=sapply(int3000@polygons, FUN=function(x) {slot(x, 'area')}))

row.names(area3000) <- sapply(int3000@polygons, FUN=function(x) {slot(x, 'ID')})

attArea3000 <- spCbind(int3000, area3000)

agg3000<-aggregate(area~attArea3000@data$Class+attArea3000@data$Point.No, data=attArea3000, FUN=sum)

write.csv(agg250, file="C:/Users/rebecca.trubitt/Desktop/ResearchR/data/area250_date.csv") #Change parameters to save new file for each buffer

##Find landscape level tree edge: Import new shapefile (only tree cover), re-do clips with only trees, calculate and aggregate shape perimeter

tree<-readOGR(dsn= "C:/Users/rebecca.trubitt/Desktop/Thesis GIS", layer= "TreeCover")

tree<- gBuffer(tree, byid=TRUE, width=0)

int250<-intersect(buff250, tree)

int500<-intersect(buff500, tree)

int1000<-intersect(buff1000, tree)

int3000<-intersect(buff3000, tree)

per250 <- data.frame(gLength(int250, byid=TRUE))

rownames(per250)<-sapply(int250@polygons, FUN=function(x) {slot(x, 'ID')})

attPer250 <- spCbind(int250, per250)

aggper250<-aggregate(attPer250@data$gLength.int250..byid...TRUE.~attPer250@data$Class+attPer250@data$Point.No, data=attPer250, FUN=sum)

write.csv(aggper250, file="C:/Users/rebecca.trubitt/Desktop/ResearchR/data/edge250_date.csv")

per500 <- data.frame(gLength(int500, byid=TRUE))

rownames(per500)<-sapply(int500@polygons, FUN=function(x) {slot(x, 'ID')})

attPer500 <- spCbind(int500, per500)

aggper500<-aggregate(attPer500@data$gLength.int500..byid...TRUE.~attPer500@data$Class+attPer500@data$Point.No, data=attPer500, FUN=sum)

write.csv(aggper500, file="C:/Users/rebecca.trubitt/Desktop/ResearchR/data/edge500_date.csv")

per1000 <- data.frame(gLength(int1000, byid=TRUE))

rownames(per1000)<-sapply(int1000@polygons, FUN=function(x) {slot(x, 'ID')})

attPer1000 <- spCbind(int1000, per1000)

aggper1000<-aggregate(attPer1000@data$gLength.int1000..byid...TRUE.~attPer1000@data$Class+attPer1000@data$Point.No, data=attPer1000, FUN=sum)

write.csv(aggper1000, file="C:/Users/rebecca.trubitt/Desktop/ResearchR/data/edge1000_date.csv")

per3000 <- data.frame(gLength(int3000, byid=TRUE))

rownames(per3000)<-sapply(int3000@polygons, FUN=function(x) {slot(x, 'ID')})

attPer3000 <- spCbind(int3000, per3000)

aggper3000<-aggregate(attPer3000@data$gLength.int3000..byid...TRUE.~attPer3000@data$Class+attPer3000@data$Point.No, data=attPer3000, FUN=sum)

write.csv(aggper3000, file="C:/Users/rebecca.trubitt/Desktop/ResearchR/data/edge3000_date.csv")

##Find road density with similar protocol to that for tree edge length

road<-readOGR(dsn= "C:/Users/rebecca.trubitt/Desktop/Thesis GIS/Roads", layer= "Road_combo")

int250<-intersect(road, buff250)

int500<-intersect(road, buff500)

int1000<-intersect(road, buff1000)

int3000<-intersect(road, buff3000)

rd250 <- data.frame(gLength(int250, byid=TRUE))

rownames(rd250)<-sapply(int250@lines, FUN=function(x) {slot(x, 'ID')})

attPer250 <- spCbind(int250, rd250)

aggper250<-aggregate(attPer250@data$gLength.int250..byid...TRUE.~attPer250@data$Point.No, data=attPer250, FUN=sum)

write.csv(aggper250, file="C:/Users/rebecca.trubitt/Desktop/ResearchR/data/rd250_date.csv")

rd500 <- data.frame(gLength(int500, byid=TRUE))

rownames(rd500)<-sapply(int500@lines, FUN=function(x) {slot(x, 'ID')})

attPer500 <- spCbind(int500, rd500)

aggper500<-aggregate(attPer500@data$gLength.int500..byid...TRUE.~attPer500@data$Point.No, data=attPer500, FUN=sum)

write.csv(aggper500, file="C:/Users/rebecca.trubitt/Desktop/ResearchR/data/rd500_date.csv")

rd1000 <- data.frame(gLength(int1000, byid=TRUE))

rownames(rd1000)<-sapply(int1000@lines, FUN=function(x) {slot(x, 'ID')})

attPer1000 <- spCbind(int1000, rd1000)

aggper1000<-aggregate(attPer1000@data$gLength.int1000..byid...TRUE.~attPer1000@data$Point.No, data=attPer1000, FUN=sum)

write.csv(aggper1000, file="C:/Users/rebecca.trubitt/Desktop/ResearchR/data/rd1000_date.csv")

rd3000 <- data.frame(gLength(int3000, byid=TRUE))

rownames(rd3000)<-sapply(int3000@lines, FUN=function(x) {slot(x, 'ID')})

attPer3000 <- spCbind(int3000, rd3000)

aggper3000<-aggregate(attPer3000@data$gLength.int3000..byid...TRUE.~attPer3000@data$Point.No, data=attPer3000, FUN=sum)

write.csv(aggper3000, file="C:/Users/rebecca.trubitt/Desktop/ResearchR/data/rd3000_date.csv")

##Additional variables (Distance to tree, distance to open water, distance to human built structure) were measured manually in ArcGIS.

##There is also a 'distance to' protocol in ArcGIS that you can use if you have a shapefile of the landscape feature in question.

#Step 3: Use vifmer to check for collinearity between predictor variables

##vif.mer- This function calculates Variance Inflation Factors for each variable in a model. I required VIF factors to be lower than 3, following

##the protocol suggested by Zuur et al, 2009. The function for vif.mer (vif function for mixed models) is from https://github.com/aufrank/R-hacks/blob/master/mer-utils.R.

vif.mer <- function (fit) {

## adapted from rms::vif

v <- vcov(fit)

nam <- names(fixef(fit))

## exclude intercepts

ns <- sum(1 * (nam == "Intercept" | nam == "(Intercept)"))

if (ns > 0) {

v <- v[-(1:ns), -(1:ns), drop = FALSE]

nam <- nam[-(1:ns)]

}

d <- diag(v)^0.5

v <- diag(solve(v/(d %o% d)))

names(v) <- nam

v

}

LN.global.250<-glmer(LanoMin ~ PZTree+PZWater+PZStruct+LZTree250+LZWater250+LZWetland250+LZCrop250+LZRatio250+LZRoad250+(1|Detector), data=act, family=poisson)

LN.global.500<-glmer(LanoMin ~ PZTree+PZWater+PZStruct+LZTree500+LZWater500+LZWetland500+LZCrop500+LZRatio500+LZRoad500+(1|Detector), data=act, family=poisson)

LN.global.1000<-glmer(LanoMin ~ PZTree+PZWater+PZStruct+LZTree1000+LZWater1000+LZWetland1000+LZCrop1000+LZRatio1000+LZRoad1000+(1|Detector), data=act, family=poisson)

vif.mer(LN.global.1000)

LC.global.250<-glmer(LaciMin ~ PZTree+PZWater+PZStruct+LZTree250+LZWater250+LZWetland250+LZCrop250+LZRatio250+LZRoad250+(1|Detector), data=act, family=poisson)

LC.global.500<-glmer(LaciMin ~ PZTree+PZWater+PZStruct+LZTree500+LZWater500+LZWetland500+LZCrop500+LZRatio500+LZRoad500+(1|Detector), data=act, family=poisson)

LC.global.1000<-glmer(LaciMin ~ PZTree+PZWater+PZStruct+LZTree1000+LZWater1000+LZWetland1000+LZCrop1000+LZRatio1000+LZRoad1000+(1|Detector), data=act, family=poisson)

vif.mer(LC.global.1000)

EF.global.250<-glmer(EpfuMin ~ PZTree+PZWater+PZStruct+LZTree250+LZWater250+LZWetland250+LZCrop250+LZRatio250+LZRoad250+(1|Detector), data=act, family=poisson)

EF.global.500<-glmer(EpfuMin ~ PZTree+PZWater+PZStruct+LZTree500+LZWater500+LZWetland500+LZCrop500+LZRatio500+LZRoad500+(1|Detector), data=act, family=poisson)

EF.global.1000<-glmer(EpfuMin ~ PZTree+PZWater+PZStruct+LZTree1000+LZWater1000+LZWetland1000+LZCrop1000+LZRatio1000+LZRoad1000+(1|Detector), data=act, family=poisson)

vif.mer(EF.global.1000)

#Step 4: Run GLMMs and compare using AIC (included in model output). Originally, models were built with lme4 and the Poisson family. However, due to

#overdistribution in these models, we used the negative binomial family in the package glmmTMB instead. These models are shown below.

##L. noctivagans

LN.global.250<-glmmTMB(LanoMin ~ PZTree+PZWater+PZStruct+LZTree250+LZWater250+LZWetland250+LZCrop250+LZRatio250+LZRoad250+(1|Detector), data=act, family="nbinom2")

LN.landscape.250<-glmmTMB(LanoMin ~ LZTree250+LZWater250+LZWetland250+LZCrop250+LZRatio250+LZRoad250+(1|Detector), data=act, family="nbinom2")

LN.landcover.250<-glmmTMB(LanoMin ~ LZTree250+LZWater250+LZWetland250+LZCrop250+(1|Detector), data=act, family="nbinom2")

LN.proximate.250<-glmmTMB(LanoMin ~ PZTree+PZWater+PZStruct+(1|Detector), data=act, family="nbinom2")

LN.roost.250<-glmmTMB(LanoMin ~ PZTree+PZStruct+LZTree250+(1|Detector), data=act, family="nbinom2")

LN.tree.250<-glmmTMB(LanoMin ~ PZTree+LZTree250+LZRatio250+(1|Detector), data=act, family="nbinom2")

LN.water.250<-glmmTMB(LanoMin ~ PZWater+LZWater250+LZWetland250+(1|Detector), data=act, family="nbinom2")

LN.devo.250<-glmmTMB(LanoMin ~ PZStruct+LZCrop250+LZRoad250+(1|Detector), data=act, family="nbinom2")

LN.null.250<-glmmTMB(LanoMin ~ 1+(1|Detector), data=act, family="nbinom2")

##chooses landcover and roost

LN.global.500<-glmmTMB(LanoMin ~ PZTree+PZWater+PZStruct+LZTree500+LZWater500+LZWetland500+LZCrop500+LZRatio500+LZRoad500+(1|Detector), data=act, family="nbinom2")

LN.landscape.500<-glmmTMB(LanoMin ~ LZTree500+LZWater500+LZWetland500+LZCrop500+LZRatio500+LZRoad500+(1|Detector), data=act, family="nbinom2")

LN.landcover.500<-glmmTMB(LanoMin ~ LZTree500+LZWater500+LZWetland500+LZCrop500+(1|Detector), data=act, family="nbinom2")

LN.proximate.500<-glmmTMB(LanoMin ~ PZTree+PZWater+PZStruct+(1|Detector), data=act, family="nbinom2")

LN.roost.500<-glmmTMB(LanoMin ~ PZTree+PZStruct+LZTree500+(1|Detector), data=act, family="nbinom2")

LN.tree.500<-glmmTMB(LanoMin ~ PZTree+LZTree500+LZRatio500+(1|Detector), data=act, family="nbinom2")

LN.water.500<-glmmTMB(LanoMin ~ PZWater+LZWater500+LZWetland500+(1|Detector), data=act, family="nbinom2")

LN.devo.500<-glmmTMB(LanoMin ~ PZStruct+LZCrop500+LZRoad500+(1|Detector), data=act, family="nbinom2")

LN.null.500<-glmmTMB(LanoMin ~ 1+(1|Detector), data=act, family="nbinom2")

##chooses tree, roost, landcover, landscape

LN.global.1000<-glmmTMB(LanoMin ~ PZTree+PZWater+PZStruct+LZTree1000+LZWater1000+LZWetland1000+LZCrop1000+LZRatio1000+LZRoad1000+(1|Detector), data=act, family="nbinom2")

LN.landscape.1000<-glmmTMB(LanoMin ~ LZTree1000+LZWater1000+LZWetland1000+LZCrop1000+LZRatio1000+LZRoad1000+(1|Detector), data=act, family="nbinom2")

LN.landcover.1000<-glmmTMB(LanoMin ~ LZTree1000+LZWater1000+LZWetland1000+LZCrop1000+(1|Detector), data=act, family="nbinom2")

LN.proximate.1000<-glmmTMB(LanoMin ~ PZTree+PZWater+PZStruct+(1|Detector), data=act, family="nbinom2")

LN.roost.1000<-glmmTMB(LanoMin ~ PZTree+PZStruct+LZTree1000+(1|Detector), data=act, family="nbinom2")

LN.tree.1000<-glmmTMB(LanoMin ~ PZTree+LZTree1000+LZRatio1000+(1|Detector), data=act, family="nbinom2")

LN.water.1000<-glmmTMB(LanoMin ~ PZWater+LZWater1000+LZWetland1000+(1|Detector), data=act, family="nbinom2")

LN.devo.1000<-glmmTMB(LanoMin ~ PZStruct+LZCrop1000+LZRoad1000+(1|Detector), data=act, family="nbinom2")

LN.null.1000<-glmmTMB(LanoMin ~ 1+(1|Detector), data=act, family="nbinom2")

##Chooses water, roost and landcover

##Top models are 500m tree, roost, landcover and landscape

##L. cinereus

LC.global.250<-glmmTMB(LaciMin ~ PZTree+PZWater+PZStruct+LZTree250+LZWater250+LZWetland250+LZCrop250+LZRatio250+LZRoad250+(1|Detector), data=act, family="nbinom2")

LC.landscape.250<-glmmTMB(LaciMin ~ LZTree250+LZWater250+LZWetland250+LZCrop250+LZRatio250+LZRoad250+(1|Detector), data=act, family="nbinom2")

LC.landcover.250<-glmmTMB(LaciMin ~ LZTree250+LZWater250+LZWetland250+LZCrop250+(1|Detector), data=act, family="nbinom2")

LC.proximate.250<-glmmTMB(LaciMin ~ PZTree+PZWater+PZStruct+(1|Detector), data=act, family="nbinom2")

LC.roost.250<-glmmTMB(LaciMin ~ PZTree+LZTree250+(1|Detector), data=act, family="nbinom2")

LC.tree.250<-glmmTMB(LaciMin ~ PZTree+LZTree250+LZRatio250+(1|Detector), data=act, family="nbinom2")

LC.water.250<-glmmTMB(LaciMin ~ PZWater+LZWater250+LZWetland250+(1|Detector), data=act, family="nbinom2")

LC.devo.250<-glmmTMB(LaciMin ~ PZStruct+LZCrop250+LZRoad250+(1|Detector), data=act, family="nbinom2")

LC.null.250<-glmmTMB(LaciMin ~ 1+(1|Detector), data=act, family="nbinom2")

##Chooses water, global

LC.global.500<-glmmTMB(LaciMin ~ PZTree+PZWater+PZStruct+LZTree500+LZWater500+LZWetland500+LZCrop500+LZRatio500+LZRoad500+(1|Detector), data=act, family="nbinom2")

LC.landscape.500<-glmmTMB(LaciMin ~ LZTree500+LZWater500+LZWetland500+LZCrop500+LZRatio500+LZRoad500+(1|Detector), data=act, family="nbinom2")

LC.landcover.500<-glmmTMB(LaciMin ~ LZTree500+LZWater500+LZWetland500+LZCrop500+(1|Detector), data=act, family="nbinom2")

LC.proximate.500<-glmmTMB(LaciMin ~ PZTree+PZWater+PZStruct+(1|Detector), data=act, family="nbinom2")

LC.roost.500<-glmmTMB(LaciMin ~ PZTree+LZTree500+(1|Detector), data=act, family="nbinom2")

LC.tree.500<-glmmTMB(LaciMin ~ PZTree+LZTree500+LZRatio500+(1|Detector), data=act, family="nbinom2")

LC.water.500<-glmmTMB(LaciMin ~ PZWater+LZWater500+LZWetland500+(1|Detector), data=act, family="nbinom2")

LC.devo.500<-glmmTMB(LaciMin ~ PZStruct+LZCrop500+LZRoad500+(1|Detector), data=act, family="nbinom2")

LC.null.500<-glmmTMB(LaciMin ~ 1+(1|Detector), data=act, family="nbinom2")

##Chooses global and water

LC.global.1000<-glmmTMB(LaciMin ~ PZTree+PZWater+PZStruct+LZTree1000+LZWater1000+LZWetland1000+LZCrop1000+LZRatio1000+LZRoad1000+(1|Detector), data=act, family="nbinom2")

LC.landscape.1000<-glmmTMB(LaciMin ~ LZTree1000+LZWater1000+LZWetland1000+LZCrop1000+LZRatio1000+LZRoad1000+(1|Detector), data=act, family="nbinom2")

LC.landcover.1000<-glmmTMB(LaciMin ~ LZTree1000+LZWater1000+LZWetland1000+LZCrop1000+(1|Detector), data=act, family="nbinom2")

LC.proximate.1000<-glmmTMB(LaciMin ~ PZTree+PZWater+PZStruct+(1|Detector), data=act, family="nbinom2")

LC.roost.1000<-glmmTMB(LaciMin ~ PZTree+LZTree1000+(1|Detector), data=act, family="nbinom2")

LC.tree.1000<-glmmTMB(LaciMin ~ PZTree+LZTree1000+LZRatio1000+(1|Detector), data=act, family="nbinom2")

LC.water.1000<-glmmTMB(LaciMin ~ PZWater+LZWater1000+LZWetland1000+(1|Detector), data=act, family="nbinom2")

LC.devo.1000<-glmmTMB(LaciMin ~ PZStruct+LZCrop1000+LZRoad1000+(1|Detector), data=act, family="nbinom2")

LC.null.1000<-glmmTMB(LaciMin ~ 1+(1|Detector), data=act, family="nbinom2")

##Chooses water, global

##Top models are 500m global and water and 1000m global and water.

##E. fuscus

EF.global.250<-glmmTMB(EpfuMin ~ PZTree+PZWater+PZStruct+LZTree250+LZWater250+LZWetland250+LZCrop250+LZRatio250+LZRoad250+(1|Detector), data=act, family="nbinom2")

EF.landscape.250<-glmmTMB(EpfuMin ~ LZTree250+LZWater250+LZWetland250+LZCrop250+LZRatio250+LZRoad250+(1|Detector), data=act, family="nbinom2")

EF.landcover.250<-glmmTMB(EpfuMin ~ LZTree250+LZWater250+LZWetland250+LZCrop250+(1|Detector), data=act, family="nbinom2")

EF.proximate.250<-glmmTMB(EpfuMin ~ PZTree+PZWater+PZStruct+(1|Detector), data=act, family="nbinom2")

EF.roost.250<-glmmTMB(EpfuMin ~ PZTree+PZStruct+LZTree250+(1|Detector), data=act, family="nbinom2")

EF.tree.250<-glmmTMB(EpfuMin ~ PZTree+LZTree250+LZRatio250+(1|Detector), data=act, family="nbinom2")

EF.water.250<-glmmTMB(EpfuMin ~ PZWater+LZWater250+LZWetland250+(1|Detector), data=act, family="nbinom2")

EF.devo.250<-glmmTMB(EpfuMin ~ PZStruct+LZCrop250+LZRoad250+(1|Detector), data=act, family="nbinom2")

EF.null.250<-glmmTMB(EpfuMin ~ 1+(1|Detector), data=act, family="nbinom2")

##Chooses global, water, and landscape

EF.global.500<-glmmTMB(EpfuMin ~ PZTree+PZWater+PZStruct+LZTree500+LZWater500+LZWetland500+LZCrop500+LZRatio500+LZRoad500+(1|Detector), data=act, family="nbinom2")

EF.landscape.500<-glmmTMB(EpfuMin ~ LZTree500+LZWater500+LZWetland500+LZCrop500+LZRatio500+LZRoad500+(1|Detector), data=act, family="nbinom2")

EF.landcover.500<-glmmTMB(EpfuMin ~ LZTree500+LZWater500+LZWetland500+LZCrop500+(1|Detector), data=act, family="nbinom2")

EF.proximate.500<-glmmTMB(EpfuMin ~ PZTree+PZWater+PZStruct+(1|Detector), data=act, family="nbinom2")

EF.roost.500<-glmmTMB(EpfuMin ~ PZTree+PZStruct+LZTree500+(1|Detector), data=act, family="nbinom2")

EF.tree.500<-glmmTMB(EpfuMin ~ PZTree+LZTree500+LZRatio500+(1|Detector), data=act, family="nbinom2")

EF.water.500<-glmmTMB(EpfuMin ~ PZWater+LZWater500+LZWetland500+(1|Detector), data=act, family="nbinom2")

EF.devo.500<-glmmTMB(EpfuMin ~ PZStruct+LZCrop500+LZRoad500+(1|Detector), data=act, family="nbinom2")

EF.null.500<-glmmTMB(EpfuMin ~ 1+(1|Detector), data=act, family="nbinom2")

##Chooses roost, water, tree, landcover, proximate

EF.global.1000<-glmmTMB(EpfuMin ~ PZTree+PZWater+PZStruct+LZTree1000+LZWater1000+LZWetland1000+LZCrop1000+LZRatio1000+LZRoad1000+(1|Detector), data=act, family="nbinom2")

EF.landscape.1000<-glmmTMB(EpfuMin ~ LZTree1000+LZWater1000+LZWetland1000+LZCrop1000+LZRatio1000+LZRoad1000+(1|Detector), data=act, family="nbinom2")

EF.landcover.1000<-glmmTMB(EpfuMin ~ LZTree1000+LZWater1000+LZWetland1000+LZCrop1000+(1|Detector), data=act, family="nbinom2")

EF.proximate.1000<-glmmTMB(EpfuMin ~ PZTree+PZWater+PZStruct+(1|Detector), data=act, family="nbinom2")

EF.roost.1000<-glmmTMB(EpfuMin ~ PZTree+PZStruct+LZTree1000+(1|Detector), data=act, family="nbinom2")

EF.tree.1000<-glmmTMB(EpfuMin ~ PZTree+LZTree1000+LZRatio1000+(1|Detector), data=act, family="nbinom2")

EF.water.1000<-glmmTMB(EpfuMin ~ PZWater+LZWater1000+LZWetland1000+(1|Detector), data=act, family="nbinom2")

EF.devo.1000<-glmmTMB(EpfuMin ~ PZStruct+LZCrop1000+LZRoad1000+(1|Detector), data=act, family="nbinom2")

EF.null.1000<-glmmTMB(EpfuMin ~ 1+(1|Detector), data=act, family="nbinom2")

##Chooses global, landscape.

##Top models are 1000m landscape and global

##L. borealis

LB.global.250<-glmmTMB(LaboMin ~ PZTree+PZWater+PZStruct+LZTree250+LZWater250+LZWetland250+LZCrop250+LZRatio250+LZRoad250+(1|Detector), data=act, family="nbinom2")

LB.landscape.250<-glmmTMB(LaboMin ~ LZTree250+LZWater250+LZWetland250+LZCrop250+LZRatio250+LZRoad250+(1|Detector), data=act, family="nbinom2")

LB.landcover.250<-glmmTMB(LaboMin ~ LZTree250+LZWater250+LZWetland250+LZCrop250+(1|Detector), data=act, family="nbinom2")

LB.proximate.250<-glmmTMB(LaboMin ~ PZTree+PZWater+PZStruct+(1|Detector), data=act, family="nbinom2")

LB.roost.250<-glmmTMB(LaboMin ~ PZTree+LZTree250+(1|Detector), data=act, family="nbinom2")

LB.tree.250<-glmmTMB(LaboMin ~ PZTree+LZTree250+LZRatio250+(1|Detector), data=act, family="nbinom2")

LB.water.250<-glmmTMB(LaboMin~ PZWater+LZWater250+LZWetland250+(1|Detector), data=act, family="nbinom2")

LB.devo.250<-glmmTMB(LaboMin ~ PZStruct+LZCrop250+LZRoad250+(1|Detector), data=act, family="nbinom2")

LB.null.250<-glmmTMB(LaboMin ~ 1+(1|Detector), data=act, family="nbinom2")

##Chooses roost and tree

LB.global.500<-glmmTMB(LaboMin ~ PZTree+PZWater+PZStruct+LZTree500+LZWater500+LZWetland500+LZCrop500+LZRatio500+LZRoad500+(1|Detector), data=act, family="nbinom2")

LB.landscape.500<-glmmTMB(LaboMin ~ LZTree500+LZWater500+LZWetland500+LZCrop500+LZRatio500+LZRoad500+(1|Detector), data=act, family="nbinom2")

LB.landcover.500<-glmmTMB(LaboMin ~ LZTree500+LZWater500+LZWetland500+LZCrop500+(1|Detector), data=act, family="nbinom2")

LB.proximate.500<-glmmTMB(LaboMin ~ PZTree+PZWater+PZStruct+(1|Detector), data=act, family="nbinom2")

LB.roost.500<-glmmTMB(LaboMin ~ PZTree+LZTree500+(1|Detector), data=act, family="nbinom2")

LB.tree.500<-glmmTMB(LaboMin ~ PZTree+LZTree500+LZRatio500+(1|Detector), data=act, family="nbinom2")

LB.water.500<-glmmTMB(LaboMin ~ PZWater+LZWater500+LZWetland500+(1|Detector), data=act, family="nbinom2")

LB.devo.500<-glmmTMB(LaboMin ~ PZStruct+LZCrop500+LZRoad500+(1|Detector), data=act, family="nbinom2")

LB.null.500<-glmmTMB(LaboMin ~ 1+(1|Detector), data=act, family="nbinom2")

##Chooses roost and tree

confint(LB.tree.500)

LB.global.1000<-glmmTMB(LaboMin ~ PZTree+PZWater+PZStruct+LZTree1000+LZWater1000+LZWetland1000+LZCrop1000+LZRatio1000+LZRoad1000+(1|Detector), data=act, family="nbinom2")

LB.landscape.1000<-glmmTMB(LaboMin ~ LZTree1000+LZWater1000+LZWetland1000+LZCrop1000+LZRatio1000+LZRoad1000+(1|Detector), data=act, family="nbinom2")

LB.landcover.1000<-glmmTMB(LaboMin ~ LZTree1000+LZWater1000+LZWetland1000+LZCrop1000+(1|Detector), data=act, family="nbinom2")

LB.proximate.1000<-glmmTMB(LaboMin ~ PZTree+PZWater+PZStruct+(1|Detector), data=act, family="nbinom2")

LB.roost.1000<-glmmTMB(LaboMin ~ PZTree+LZTree1000+(1|Detector), data=act, family="nbinom2")

LB.tree.1000<-glmmTMB(LaboMin ~ PZTree+LZTree1000+LZRatio1000+(1|Detector), data=act, family="nbinom2")

LB.water.1000<-glmmTMB(LaboMin ~ PZWater+LZWater1000+LZWetland1000+(1|Detector), data=act, family="nbinom2")

LB.devo.1000<-glmmTMB(LaboMin ~ PZStruct+LZCrop1000+LZRoad1000+(1|Detector), data=act, family="nbinom2")

LB.null.1000<-glmmTMB(LaboMin ~ 1+(1|Detector), data=act, family="nbinom2")

##Chooses roost and tree

##Top models are 250m roost and 500m roost and tree

#Step 5: Determine coefficients and confidence intervals for top models

LN.global.500

confint(LN.global.500)

##repeat with all top models

#Step 6: Graph

lanocoef<-read.csv("E:/BeckysStuff/LANOtable2.csv")

lanocoef$Var<- factor(lanocoef$Var, as.character(lanocoef$Var))

LANO <- ggplot(lanocoef, aes(x=Var, y=LANO, shape=Model)) +

geom_point(stat="identity", position = position_dodge(width = .6), size=3) + theme_bw()+

geom_hline(yintercept=0, colour="red")+

geom_errorbar(aes(ymin=CIL,ymax=CIU), position = position_dodge(width = .6), width=0.3)+

scale_x_discrete(name="Model Term")+

labs(title="L. noctivagans", y="Coefficients")+

theme(axis.text.y = element_text(size="16", vjust=0.6), axis.text.x= element_text(size="15"), plot.title=element_text(size="18"),legend.text=element_text(size="14"))+

expand_limits(y=c(-0.8, 0.8))+

coord_flip()

LANO

lacicoef<-read.csv("E:/BeckysStuff/LACItable2.csv")

lacicoef$Var<- factor(lacicoef$Var, as.character(lacicoef$Var))

LACI <- ggplot(lacicoef, aes(x=Var, y=LACI, shape=Model)) +

geom_point(stat="identity", position = position_dodge(width = .6), size=3) + theme_bw()+

geom_hline(yintercept=0, colour="red")+

geom_errorbar(aes(ymin=CIL,ymax=CIU), position = position_dodge(width = .6), width=0.3)+

scale_x_discrete(name="Model Term")+

labs(title="L. cinereus", y="Coefficients")+

theme(axis.text.y = element_text(size="16", vjust=0.6), axis.text.x= element_text(size="15"), plot.title=element_text(size="18"),legend.text=element_text(size="14"))+

expand_limits(y=c(-0.8, 0.8))+

coord_flip()

LACI

epfucoef<-read.csv("E:/BeckysStuff/EPFUtable2.csv")

epfucoef$Var<- factor(epfucoef$Var, as.character(epfucoef$Var))

EPFU <- ggplot(epfucoef, aes(x=Var, y=EPFU, shape=Model)) +

geom_point(stat="identity", position = position_dodge(width = .6), size=3) + theme_bw()+

geom_hline(yintercept=0, colour="red")+

geom_errorbar(aes(ymin=CIL,ymax=CIU), position = position_dodge(width = .6), width=0.3)+

scale_x_discrete(name="Model Term")+

labs(title="E. fuscus", y="Coefficients")+

theme(axis.text.y = element_text(size="16", vjust=0.6), axis.text.x= element_text(size="15"), plot.title=element_text(size="18"),legend.text=element_text(size="14"))+

expand_limits(y=c(-0.8, 0.8))+

coord_flip()

EPFU

labocoef<-read.csv("E:/BeckysStuff/LABOtable2.csv")

labocoef$Var<- factor(labocoef$Var, as.character(labocoef$Var))

LABO <- ggplot(labocoef, aes(x=Var, y=LABO, shape=Model)) +

geom_point(stat="identity", position = position_dodge(width = .6), size=3) + theme_bw()+

geom_hline(yintercept=0, colour="red")+

geom_errorbar(aes(ymin=CIL,ymax=CIU), position = position_dodge(width = .6), width=0.3)+

scale_x_discrete(name="Model Term")+

labs(title="L. borealis", y="Coefficients")+

theme(axis.text.y = element_text(size="16", vjust=0.6), axis.text.x= element_text(size="15"), plot.title=element_text(size="18"),legend.text=element_text(size="14"))+

expand_limits(y=c(-0.8, 0.8))+

coord_flip()

LABO

x11()

grid.arrange(LANO, LACI, EPFU, LABO, ncol=2)

#Step 7: Use Moran's I to test competative models for spatial autocorrelation. Readout is commented below the model code.

residuals<-residuals(LB.tree.500)

write.csv(residuals, file="M:/EcologyAndEvo2018Manuscript/LBtree500SAC.csv")

#Append latitude and longitude in Excel.

file<-read.csv("M:/EcologyAndEvo2018Manuscript/LBtree500SAC.csv")

distance<-as.matrix(dist(cbind(file$Long, file$Lat)))

distance.inv <- 1/ln.dist

diag(distance.inv) <- 0

Moran.I(file$x, distance.inv)

LN.landscape.500<-glmmTMB(LanoMin ~ LZTree500+LZWater500+LZWetland500+LZCrop500+LZRatio500+LZRoad500+(1|Detector), data=act, family="nbinom2")

#$`observed`

#[1] -0.001670777

#$expected

#[1] -0.004237288

#$sd

#[1] 0.00741993

#$p.value

#[1] 0.7294222

LN.landcover.500<-glmmTMB(LanoMin ~ LZTree500+LZWater500+LZWetland500+LZCrop500+(1|Detector), data=act, family="nbinom2")

#$`observed`

#[1] -0.005227457

#$expected

#[1] -0.004237288

#$sd

#[1] 0.007394362

#$p.value

#[1] 0.8934748

LN.roost.500<-glmmTMB(LanoMin ~ PZTree+PZStruct+LZTree500+(1|Detector), data=act, family="nbinom2")

#$`observed`

#[1] 0.0007209456

#$expected

#[1] -0.004237288

#$sd

#[1] 0.007425557

#$p.value

#[1] 0.5043088

LN.tree.500<-glmmTMB(LanoMin ~ PZTree+LZTree500+LZRatio500+(1|Detector), data=act, family="nbinom2")

#$`observed`

#[1] 0.002000552

#$expected

#[1] -0.004237288

#$sd

#[1] 0.00744504

#$p.value

#[1] 0.4021139

LC.global.500<-glmmTMB(LaciMin ~ PZTree+PZWater+PZStruct+LZTree500+LZWater500+LZWetland500+LZCrop500+LZRatio500+LZRoad500+(1|Detector), data=act, family="nbinom2")

#$`observed`

#[1] -0.004863336

#$expected

#[1] -0.004237288

#$sd

#[1] 0.006648392

#$p.value

#[1] 0.9249778

LC.water.500<-glmmTMB(LaciMin ~ PZWater+LZWater500+LZWetland500+(1|Detector), data=act, family="nbinom2")

#$`observed`

#[1] -0.006904458

#$expected

#[1] -0.004237288

#$sd

#[1] 0.006536525

#$p.value

#[1] 0.6832436

LC.global.1000<-glmmTMB(LaciMin ~ PZTree+PZWater+PZStruct+LZTree1000+LZWater1000+LZWetland1000+LZCrop1000+LZRatio1000+LZRoad1000+(1|Detector), data=act, family="nbinom2")

#$`observed`

#[1] -0.009547859

#$expected

#[1] -0.004237288

#$sd

#[1] 0.006534756

#$p.value

#[1] 0.4164099

LC.water.1000<-glmmTMB(LaciMin ~ PZWater+LZWater1000+LZWetland1000+(1|Detector), data=act, family="nbinom2")

#$`observed`

#[1] -0.00818532

#$expected

#[1] -0.004237288

#$sd

#[1] 0.006413582

#$p.value

#[1] 0.538176

EF.global.1000<-glmmTMB(EpfuMin ~ PZTree+PZWater+PZStruct+LZTree1000+LZWater1000+LZWetland1000+LZCrop1000+LZRatio1000+LZRoad1000+(1|Detector), data=act, family="nbinom2")

#$`observed`

#[1] 0.003772003

#$expected

#[1] -0.004237288

#$sd

#[1] 0.007056681

#$p.value

#[1] 0.2563778

EF.landscape.1000<-glmmTMB(EpfuMin ~ LZTree1000+LZWater1000+LZWetland1000+LZCrop1000+LZRatio1000+LZRoad1000+(1|Detector), data=act, family="nbinom2")

#$`observed`

#[1] 0.002188917

#$expected

#[1] -0.004237288

#$sd

#[1] 0.007075571

#$p.value

#[1] 0.3637597

LB.roost.250<-glmmTMB(LaboMin ~ PZTree+LZTree250+(1|Detector), data=act, family="nbinom2")

#$`observed`

#[1] -0.01231694

#$expected

#[1] -0.004237288

#$sd

#[1] 0.006584891

#$p.value

#[1] 0.219823

LB.roost.500<-glmmTMB(LaboMin ~ PZTree+LZTree500+(1|Detector), data=act, family="nbinom2")

#$`observed`

#[1] -0.01427485

#$expected

#[1] -0.004237288

#$sd

#[1] 0.006592218

#$p.value

#[1] 0.1278493

LB.tree.500<-glmmTMB(LaboMin ~ PZTree+LZTree500+LZRatio500+(1|Detector), data=act, family="nbinom2")

#$`observed`

#[1] -0.01448648

#$expected

#[1] -0.004237288

#$sd

#[1] 0.006717392

#$p.value

#[1] 0.1270674
